# Supplementary material for: Astaxanthin-Loaded Pickering Emulsions Stabilized by Nanofibrillated Cellulose: Impact on Emulsion Characteristics, Digestion Behavior, and Bioaccessibility
Source: Polymers (Basel). 2023 Feb 11;15(4):901. doi: 10.3390/polym15040901 (PMC9959445; doi:10.3390/polym15040901)
Supplement: Supplementary file 1 [file polymers-15-00901-s001.zip › polymers-2128751-supplementary.pdf]

Supplementary Data

Table S1. Emulsion properties and stability of NFC-stabilized emulsions without astaxanthin which were measured for freshly prepared emulsions and those after stored at room temperature for one month

NFC concentration (%)	d_{32} (µm)		ζ-potential (mV)		$\eta_{a,100}$ (mPa.s)	
	Day 1	Day 30	Day 1	Day 30	Day 1	Day 30
0.3	7.37 ± 0.21a,C	7.79 ± 0.07a,C	–38.02 ± 5.89a,A	–35.5 ± 1.40a,A	36 ± 0.0008a,B	39 ± 0.0011a,B
0.5	10.43 ± 0.31a,B	11.12 ± 0.36a,B	–30.47 ± 6.67a,B	–27.93 ± 1.59b,B	78 ± 0.0022a,A	95 ± 0.0016a,A
0.7	13.33 ± 0.09a,A	14.21 ± 0.28a,A	–25.62 ± 3.64a,C	–23.00 ± 2.36a,C	84 ± 0.0048a,A	104 ± 0.0089a,A

Values were measured in triplicate and shown as mean ± standard deviation.
Different lowercase letters (a–b) in the same row resulted in a significant ($p \leq 0.05$) difference with respect to storage time. Different uppercase letters (A–C) in the same column resulted in a significant ($p \leq 0.05$) difference with respect to NFC concentration.

Table S2. Color (L^*, a^*, b^*) of NFC-stabilized emulsions without astaxanthin measured after 1 day and 30 days storage at room temperature for freshly prepared emulsions and those after stored at room temperature for one month

NFC concentration (%)	Color							
	L^* (lightness)		a^* (redness)		b^* (yellowness)		ΔE (Total color change)	
	Day 1	Day 30	Day 1	Day 30	Day 1	Day 30	Day 1	Day 30
0.3	76.23 ± 0.06a,A	74.87 ± 0.06b,A	0.17 ± 0.06a,A	–0.17 ± 0.06b,A	1.7 ± 0.10a,A	1.43 ± 0.06a,A	–	1.43 ± 0.04a,A
0.5	74.37 ± 0.06a,B	73.2 ± 0.10a,B	0.01± 0.00a,B	–0.27 ± 0.06b,AB	1.47 ± 0.06a,B	1.23 ± 0.06a,B	–	1.38 ± 0.05a,A
0.7	73.4 ± 0.10a,C	71.7 ± 0.60a,C	–0.23 ± 0.06a,C	–0.47 ± 0.06b,B	1.23 ± 0.06a,C	0.80 ± 0.10a,C	–	1.23 ± 0.06a,B

Values were measured in triplicate and shown as mean ± standard deviation.
Different lowercase letters (a–b) in the same row resulted in a significant ($p \leq 0.05$) difference with respect to storage time. Different uppercase letters (A–C) in the same column resulted in a significant ($p \leq 0.05$) difference with respect to NFC concentration.
